# Correlations of water iodine concentration to earlier goitre frequency in Sweden—an iodine sufficient country with long-term iodination of table salt

**DOI:** 10.1186/s12199-019-0821-9

**Published:** 2019-12-07

**Authors:** Sofia Manousou, Maja Stål, Robert Eggertsen, Michael Hoppe, Lena Hulthén, Helena Filipsson Nyström

**Affiliations:** 1 Sahlgrenska Academy, Institute of Medicine, University of Gothenburg, Göteborg, Sweden; 2 Department of Internal Medicine and Clinical Nutrition, Sahlgrenska Academy, University of Gothenburg, Göteborg, Sweden; 3 School of Public Health and Community Medicine, General Medicine, University of Gothenburg, Göteborg, Sweden; 4 Deparment of Endocrinology, Sahlgrenska University Hospital, Göteborg, Sweden

**Keywords:** Water, Iodine, Thyroid, Goitre, UIC, WIC

## Abstract

**Background:**

Before iodination of Swedish table salt in 1936, iodine deficiency resulting in goitre and hypothyroidism was common. Sweden has become iodine sufficient, as shown in a national survey in 2007, proving its iodination fortification programme effective for the general population. The objective of this study was to collect drinking water from water treatment plants nationally and test if water iodine concentration (WIC) correlated to urinary iodine concentration (UIC) of school-aged children in a national survey 2007 to former goitre frequency in 1929 and to thyroid volume data in 2007.

**Methods:**

In 2012, 166 treatment plants, located in 57% (166 of 290) of all Swedish municipalities, were asked to collect drinking water samples of approximately 10 ml. In 2007, tap water samples of the same volume were collected from 30 randomly selected schools for the national survey. Analysis of WIC was done in both treatment plants in 2012 (*n* = 166) and tap water in 2007 (*n* = 30). The correlation of WIC to the children’s UIC and thyroid volume after iodination was tested based on data from the national survey in 2007. The association of WIC to former goitre frequency was tested based on pre-iodination data, derived from a map of goitre frequency drawn in 1929.

**Results:**

The median WIC from water treatment plants was 4.0 μg/L (range 0–27 μg/L). WIC was similar in coastal and inland areas, for both ground and surface water.

WIC correlated with historical goitre areas and was lower in the goitre areas than in non-goitre areas (*p* < 0.001). WIC in the same municipalities as the schools correlated with the UIC of children (*p* < 0.01), but not with their thyroid volume.

**Conclusions:**

WIC still contributes to iodine nutrition in Sweden, but iodination overrides the goitre effect.

## Background

Nutritional iodine deficiency (ID) is the most common cause of goitre in the world [1]. An insufficient iodine intake may result in hypothyroidism or in autonomous areas within the thyroid gland, toxic nodular goitre (TNG) [2]. An iodine intake of 150 μg/day is a recommended intake for adults [3], and in Sweden, the most important iodine sources are iodinated salt, dairy products, fish, and seafood [4].

At the beginning of the twentieth century, ID was widespread in Sweden, with an occurrence of goitre in 18% of the population [5], and in some areas, goitre was present in 60% of the children [6]. Therefore, the Swedish national iodine fortification programme for table salt started in 1936 [7]. A national survey in 2007 showed that there was no goitre observed in schoolchildren and their median urinary iodine concentration (UIC) was 125 μg/L [8, 9]. However, even in an iodine sufficient population, certain groups, such as pregnant and lactating women, are at risk of ID because of their increased demand for iodine [10, 11].

Water iodine concentration (WIC) may affect the iodine status of a population, as water intake is considerable for an individual. In France, in mid-nineteenth century, WIC was ten times higher in Paris than in Lyon, where it was ten times higher than in the Alps. At the same time, goitre and cretinism were common in the Alps, Lyon had goitre but without cretinism, and Paris had neither ([12], only historical reference in French, Chatin A 1851). Regions with a high frequency of goitre and cretinism are typically mountain areas, rain shadow regions, and inlands [13]. In Denmark, WIC in drinking water differs between the East and the West side of the country, which corresponds to previously detected areas of mild and moderate ID [14].

Seawater is a rich iodine reservoir and has approximately a mean WIC of 50 μg/L [15]. Generally, WIC increases with proximity to the sea, which may relate to flooding by seawater leaving subsurface marine deposits during uplift, deposition of sediments, or retraction of the sea ([16], only historical reference in German, Fellenberg 1923). Moreover, iodine release from the soil depends on the rock, the acidity of the ground, and composition of the ground, which will determine whether iodine exists as iodide or iodate, which has a lower solubility in water [13].

Information on the iodine concentration in drinking water in Sweden is limited [1]. Considerable variation is found in the WIC of local water supply. WIC in a small trial varied from < 0.05 μg/L (*n* = 10), 0.05–0.09 μg/L (*n* = 6), to > 1 μg/L water (*n* = 2) [2]. In a report from Swedish National Food Agency [4], water iodine concentration is recognised as an issue that needs further investigation.

Urinary iodine concentration (UIC) in different cohorts of a normal population also varies in Sweden [17], even though thyroid volumes in children were in a report no longer regionally different [8], in contrast to a report before the era of iodination [6].

It was hypothesised that WIC was associated with the previous goitre frequency in Sweden and with recent UIC and thyroid volume. Therefore, the objective of the study was to collect drinking water from water treatment plants nationally and to test if WIC correlated to UIC of school-aged children from the national survey in 2007 to former goitre frequency in 1929 and to recent thyroid volume data in 2007.

## Methods

### Sample collection of water and urine

**Fig. 1:**
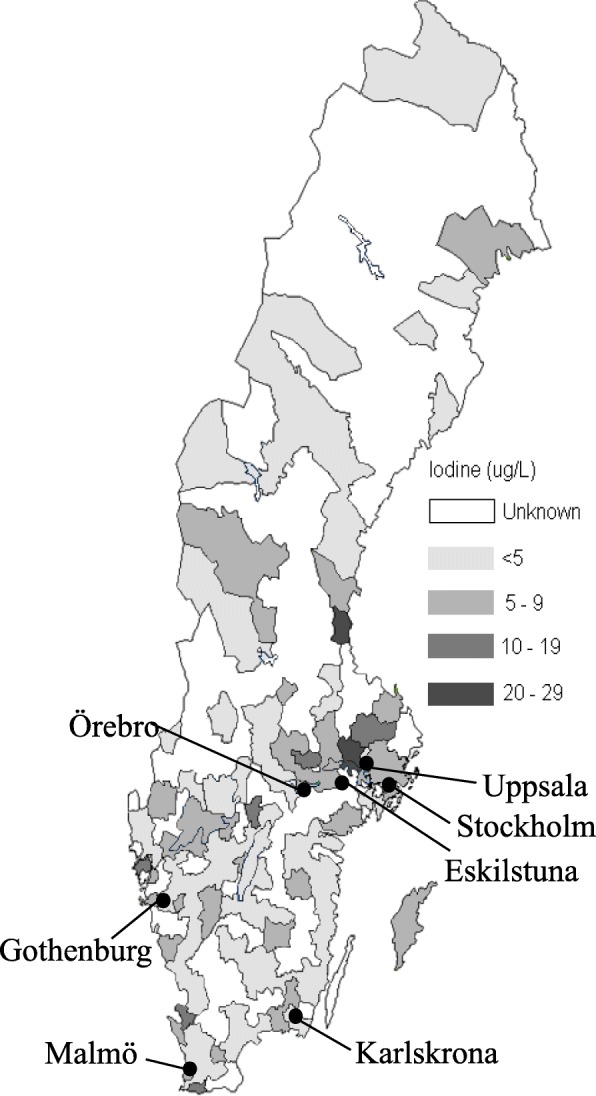
Level of water iodine concentration (WIC) from water treatment plants in 166 Swedish municipalities

**Fig. 2:**
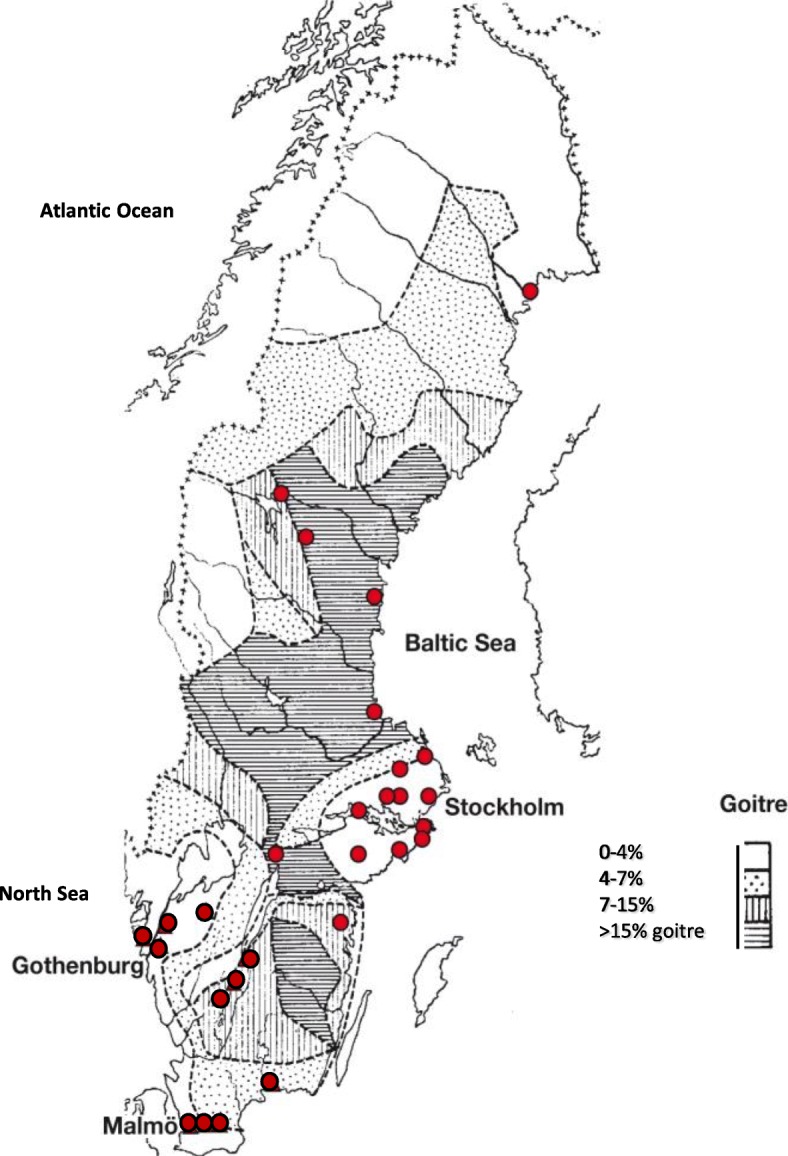
The frequency of goitre in Sweden in 1929. The dark grey shaded areas represent the goitre belt. Circles mark location of 30 randomly selected schools

All Swedish municipalities (*n* = 290) were contacted by mail in September 2012 and asked to provide water samples from their water treatment plants. There are approximately 1750 water treatment plants in Sweden. As there is often more than one water treatment plant located in each municipality, the municipalities were asked to collect water samples from their largest or larger water treatment plants and in both surface and ground water when possible (Fig. 1) and to send it to our laboratory. Two water samples of approximately 10 ml each were collected from each water treatment plant on two consecutive days from September 2012 to January 2013. Analysis of WIC was also done in earlier collected tap water samples, approximately 10 ml each, from 30 randomly selected schools in a national follow-up study of the Swedish iodine nutrition programme performed in 2006–2007 [8, 9] (Fig. 2). Each water sample was transferred in an iodine-free plastic test tube and subsequently stored at − 20°C until analysis. Urine sampling is described elsewhere [9].

### Determination of iodine concentration in water and urine

The WIC in each sample was analysed by the Sandell-Kolthoff reaction [18] at the Department of Clinical Nutrition, University of Gothenburg, Göteborg, Sweden. The laboratory participates in the EQUIP-Network (Ensuring the Quality of Urinary Iodine Procedures, Centers for Disease Control and Prevention, Atlanta, GA, USA) with success and is being evaluated for analytical accuracy every 3 months. All samples measurements were made in duplicates and re-analysed if the duplicates differed more than 2% in absorbance. The method of determination of UIC is described elsewhere [9].

### Estimation of the impact of WIC on thyroid volume

The impact of WIC on thyroid volume was estimated from data before iodination and from data after approximately 70 years of iodination. Pre-iodination data were derived from the map of goitre frequency drawn in 1929 [6]. The location of each treatment plant was marked on the map and the treatment plants were classified into four groups according to the prevalence of goitre in 1929 (Fig. 2). Modern data were retrieved from the national study in 2006–2007 that measured UIC and thyroid volumes in 857 children aged 6–12 years in 30 randomly selected schools in Sweden [8, 9]. The location of the schools was marked on the same map and the treatment plants located in the same areas as the schools were identified (Fig. 2).

### Statistics

WIC expressed as median with range (min-max). Since data were not normally distributed, differences between groups were tested by Mann-Whitney test. Urban area classifications were towns with ≥ 90,000 inhabitants and inland areas were those municipalities with no coastline to the sea. Spearman’s rank correlations were used with UIC as dependent variable and WIC as an independent variable. Pearson’s correlation was used for the evaluation of the effect from WIC on thyroid volume. The data used for the correlations are based on median UIC and median thyroid volume for each school. SPSS for Windows (version 18.0) was used for statistical calculations. A *p* value < 0.05 was considered significant.

## Results

Totally 247 of 290 Swedish communities were primary interested to deliver water samples and 57%, that is 166 with water treatment plants participated, which serve about 75% of the Swedish population with drinking water (Table 1). Most water samples came from the south and middle parts of Sweden, where the majority of the population lives, and were well distributed across these areas; fewer water samples came from the sparsely populated North part of Sweden. Most sample (74%) collections were performed during October. Some of the 166 water treatment plants delivered both ground water and surface water which is why the total number of water samples was 193 (Table 2).

**Table 1:** Median water iodine content (μg/L) in water from coastal and inland, urban and rural, and former goitrous and non-goitrous areas in ground water, surface water, and all water samples from 166 municipality water treatment plants

	*n*	All	Ground	Surface
		Median (range)	Median (range)	Median (range)
All	166	4.0	4.0	3.5
		(0–27)	(0–27)	(0–11)
Coastal	56	4.1	4.0	4.5
		(0–22)	(0–22)	(0–11)
Inland	110	3.5	4.0	3.0
		(0–27)	(0–27)	(0–13)
Urban	20	4.3	1.3*	4.5
		(0–11)	(0–11)	(0–9)
Rural	146	3.8	4.0*	3.0
		(0–27)	(0–27)	(0–27)
Goitrous	31	2.0***	2.6**	1.8***
		(0–22)	(0–22)	(0–8)
Non-goitrous	52	4.3***	6.0**	4.0***
		(0–27)	(0–27)	(0–11)

***p < 0.001, **p < 0.01, *p < 0.05 coastal vs inland, urban vs rural, and giotrous vs non-goitrous

**Table 2:** Mean (95% confidence interval) and median (interquartile range) water iodine concentration, WIC (μg/L), and number of water samples

	*n*	Mean (95% confidence interval)	Median (interquartile range)
Ground water and surface water, total	193	4.3 (3.8–4.8)	4.0 (2.0–5.9)
Ground water and surface water, coast	68	4.4 (3.5–5.3)	4.4 (1.4–6.0)
Ground water and surface water, inland	125	4.4 (3.8–5.1)	4.0 (2.0–5.0)
Surface water, coast	35	3.9 (3.0–4.9)	4.5 (1.5–6.0)
Groundwater, coast	33	4.9 (3.2–6.6)	4.0 (1.0–6.0)
Surface water, inland	62	3.4 (2.8–4.0)	3.0 (2.0–4.5)
Groundwater, inland	63	5.1 (3.9–6.2)	4.0 (2.5–6.8)
Groundwater and surface water, urban area	23	4.0 (2.8–5.2)	4.0 (2.0–5.0)
Surface water, urban area	15	4.4 (3.2–5.6)	4.5 (3.0–5.7)
Groundwater, urban area	8	2.7 (0.0–5.8)	1.3 (0.8–2.9)
Groundwater and surface water, rural area	170	4.6 (4.0–5.1)	4.0 (2.0–6.0)
Surface water, rural area	82	3.4 (2.9–4.0)	3.0 (1.5–4.5)
Groundwater, rural area	88	5.2 (4.2–6.2)	4.0 (2.4–7.1)
Surface water, total	97	3.6 (3.0–4.1)	3.5 (1.5–4.5)
Groundwater, total	96	5.0 (4.1–5.9)	4.0 (2.0–6.6)

### Water iodine concentration in drinking water in Sweden

The median WIC in all treatment plants was 4.0 μg/L and varied from 0 to 27 μg/L. Most (74%) had a WIC between 0 and 5 μg/L. WICs > 5 μg/L were often found in the south (Skåne), west (around Gothenburg) and east coast (around Stockholm) (Fig. 1). The median WIC was 4.0 μg/L (range 0–27) in ground water and 3.5 μg/L (range 0–13) in surface water (NS, Table 1, Table 2).

### WIC in coast and inland areas

Total median WIC in coastal areas (4.4 μg/L, *n* = 68) did not differ statistically from inland areas (4.0 μg/L, *p* = 0.631, *n* = 125), (Table 2). Neither were there any differences in ground water median WIC between coastal and inland areas or surface water (Table 1). Median WIC in surface water in coastal areas was 4.5 μg/L (range 0–11, *n* = 35) compared to inland areas 3.0 μg/L (range 0–13, *n* = 62, *p* = 0.267) (Table 2).

### WIC in urban and rural areas

Ground water from urban areas (1.3 μg/L, *n* = 8) had lower median WIC than ground water from regions with smaller towns (rural areas) (4.0 μg/L, *n* = 88, *p* < 0.05) (Table 2). The difference was diminished and not significant for median WIC between surface water from urban areas (4.5 μg/L, *n* = 15) and surface water from rural areas (3.0 μg/L, *n* = 82) (Table 2).

### WIC in former goitre and non-goitre areas

**Fig. 3:**
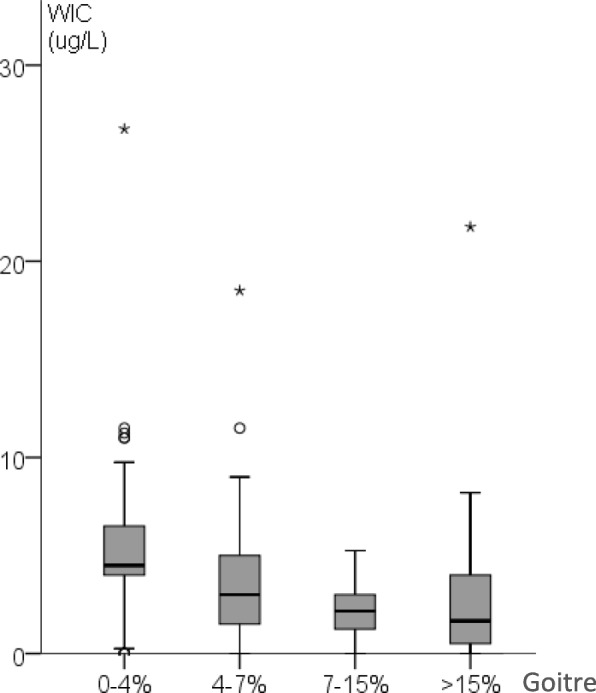
Relationship between water iodine concentration (WIC) and frequency of goitre in 1929, > 15% goitre vs non-goitre areas (*p* = 0.001). ^o^outlier, i.e. value that is 1.5–3 times the interquartile range (IQR) higher than the third quartile. ^*^extreme value, i.e. value that is more than 3 times the IQR higher than the third quartile

WIC from areas within the former Swedish goitre belt (goitre frequency > 15%) (Fig. 2), which existed before iodination of table salt and decided geographically in 1929, had lower median WIC than in non-goitre areas (goitre frequency 0–4%) (Fig. 2) (*p* < 0.001) [6] (Table 1, (Fig. 3)).

### The influence of WIC on UIC and thyroid volumes of Swedish schoolchildren during national iodination

**Fig. 4:**
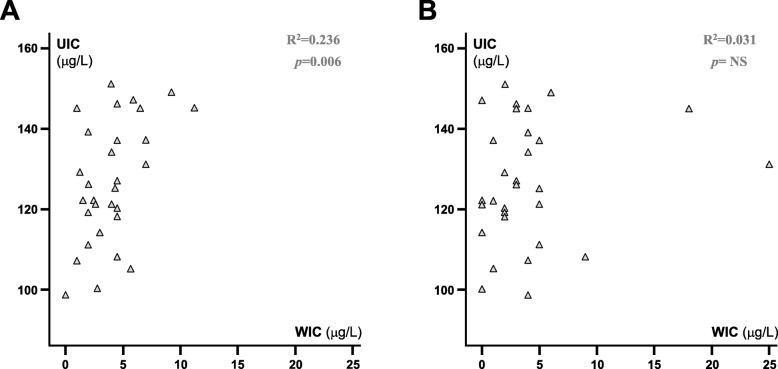
Correlation between median urinary iodine concentration (UIC) from children in each of 30 schools (Fig. 2) and median water iodine concentration (WIC) from (**a**) water treatment plants in the same Swedish municipalities (*p* = 0.006) and (**b**) the tap water in the same schools (NS)

WIC in the water samples from the same municipalities as the selected schools correlated with the UIC in children (*p* < 0.01: Fig. 4a) [9]. However, the WIC from water samples collected from tap water in the same school buildings did not correlate to the UIC of the schoolchildren (Fig. 4b) [9]. WIC in the same municipalities as the schools did not correlate to thyroid volumes in any age category of the children (data not shown) [8].

Thyroid volumes, from earlier work [8], were positively correlated with age, body surface area, weight, body mass index, and height for both boys and girls (*p* < 0.0001 for all). The most important predictors of thyroid volumes in boys were age (*R*^2^ = 0.439, *p* = 0.001), body surface area (*R*^2^ = 0.453, *p* < 0.01), and height (*R*^2^ = 0.424, *p* < 0.0001), and for girls, age (*R*^2^ = 0.596, *p* < 0.0001) and body surface area (*R*^2^ = 0.574, *p* < 0.0001).

## Discussion

The WIC from 166 Swedish municipalities’ treatment plants correlated to both the former frequency of goitre and to recent UIC in 857 schoolchildren from 30 Swedish schools, implying geographical differences in iodine intake. WIC continues to influence iodine nutrition even in a country with a long-term iodine fortification programme that results in iodine sufficiency [8, 9]. Previous problems relating to the effect of low WIC on thyroid volume were no longer evident.

Although WIC from the treatment plants of the areas nearby the schools did correlate to the children’s UIC, WIC from the tap water of the 30 schools did not. This may support the assumption that the main intake of water for an individual may be outside the schools. We could not either investigate the mid-day meal served in the schools that is prepared in different ways with or without iodized salt [9]. Surface WIC from coastal areas was comparable to surface WIC from inland areas, in contrast to the generally expected increase of surface WIC with proximity to the sea ([16], only historical reference in German, Fellenberg 1923]). On the other hand, the finding that WIC of ground water did not differ between coastal and inland areas was expected, as ground water is not influenced by aerations from sea water [13]. In Sweden, the highest iodine concentration in the ground is reported to be in the south, west, and east coast and particularly in the southwest part of the country, with iodine concentration more than 13.6 mg/kg [13].

WIC is determined in many countries. In Sweden, median WIC was 4.0 μg/L in the municipalities and ranged between 0 and 27 μg/L. This could be compared to drinking water in Denmark with a median WIC of 7.5 μg/L, range < 1–139 μg/L [19]. In Spain, WIC in drinking water is 1–20 μg/L, but the prevalence of goitre is correlated to WIC and in 1971, and the frequency of goitre increased with WIC < 10 μg/L [20], probably due to iodine deficiency at that time [21]. In a study from Venezuela [22], WIC ranged from 1 to 100 μg/L, and in Thessaloniki, Greece, WIC in tap water was 6–12 μg/L [23]. In Finland, WIC in drinking water is 0.4–9.1 μg/L [24], and in southwest Germany, WIC ranges between 0.2 and 21 μg/L [25]. Hence, the level and variation of WIC in the present study were comparable to WIC in many countries, but not all. Excessively high WIC is a problem in some areas of the world [26, 27]. In Japan, seaweed is one of the main sources of iodine intake together with foods of marine origin [28]. Our ongoing epidemiological studies may provide a reliable answer to the role of iodine in thyroid dysfunction in iodine-sufficient areas. Other studies like the Hokkaido Birth Cohort Study on environment and children’s health presented data for maternal and infant thyroid hormone levels in association with prenatal chemical exposure [29]. However, modest WIC levels such as those in Sweden may still influence iodine nutrition and thyroid outcome, as WIC correlates to UIC.

In Sweden, iodine intake from water may range between 0 and 54 μg/day if daily water intake in an adult is approximately 2 L water/day. Given that the daily recommended intake of iodine for an adult is 150 μg/day, water could represent up to one third of the recommended iodine intake in some areas of Sweden. Even if WIC influences total iodine intake, it no longer causes ID in Sweden today, as most iodine generally comes from iodinated salt, dairy products, fish, and seafood. The iodine intake from dairy products is expected to be slightly higher during the collection season compared with the summer season, as livestock are fed with iodine-enriched fodder during indoor season.

Iodinated salt contributes to daily iodine intake with 80–120 μg iodine, whereas, dairy food and seafood account for an average of 70 μg iodine per day [4]. Hence, iodinated salt, dairy food, and seafood together provide an average iodine intake of 150–190 μg per day. In the same study, the median UIC of 74 μg/L was below the WHO range of 100–200 μg/L and would thus indicate an insufficient status [30]. Using data for the creatinine-adjusted UIC (102 μg/g) and mean 24-h creatinine excretion, the median 24-h iodine excretion has been estimated to be around 130 μg. This is still below the recommended intake [3]. A decreasing trend in iodine concentration in Swedish milk has been reported, which is in line with data from Swedish Market Basket studies, indicating a decrease in the average iodine supply from food and beverage [31]. Thus, a general increase in iodine intake is desirable, especially important for women of childbearing age. The use of iodinated salt in home cooking and food products by manufacturers and increased fish consumption are feasible options. Also, efforts to restore iodine levels in milk and dairy products are warranted. However, WIC may be a determinant for iodine status if intake of iodinated salt, dairy food, and/or sea food is low, highlighting the need for coherence to an iodine fortification programme, even if it is voluntary, as in Sweden.

In regions with high WIC, iodine intake from water may be 50 μg/day for an adult, and total iodine intake may be in some individuals with a normal food consumption and use of iodinated salt as high as 200–240 μg/day. This is far below the upper limit for iodine intakes in adults: IOM (Institute of Medicine, USA) 1100 μg/day and EFSA (European Food Safety Authority) 600 μg/day. The fact is that the incidence of toxic nodular goitre will increase in ID, but when a country turns into iodine sufficiency, the rate of TNG decreases and the occurrence of autoimmune thyroid diseases (i.e., autoimmune hypothyroidism and Grave’s disease) increases [2]. More research, on the possible effect of WIC on autoimmunity in Sweden, is needed to determine whether the municipalities should define an upper limit of acceptable WIC in an iodine sufficient country.

The aim of this study was to determine WIC and correlate to UIC to previous and present thyroid outcomes. Even though the number of responding municipalities (57%) partly limited the study, the population in the participating municipalities corresponded to 75% of the Swedish population, which strengthened the study. In addition, the water samples came from municipalities representing different geological regions. Moreover, the analysis of correlation to both past and present goitre areas, and to thyroid volume in schoolchildren, provides the opportunity to discuss not only geological, but also clinical importance.

## Conclusions

This study provides new knowledge. We confirmed the hypothesis that WIC is low and that it is associated with the previous presence of goitre. WIC still contributes to iodine nutrition and is associated to UIC, but the iodination programme overrides the goitre effect. Even a low WIC will have impact on the present iodine intake and in some areas contribute to a significant proportion of the iodine intake—which may have consequences for the spectra of thyroid diseases. This is important to consider when an evaluation is made of the current iodination programme.

## Data Availability

The datasets used and/or analysed during the current study are available from the corresponding author on reasonable request.

## Abbreviations

ID: Iodine deficiency
TNG: Toxic nodular goitre
UIC: Urine iodine concentration
WIC: Water iodine concentration
